# Functional divergence of orthologous temperature-sensitive mutations in *C. elegans* and *C. briggsae*

**DOI:** 10.17912/micropub.biology.000705

**Published:** 2022-12-09

**Authors:** Satheeja Santhi Velayudhan, Ronald E Ellis

**Affiliations:** 1 Rowan University SOM

## Abstract

To learn if orthologous mutations are temperature-sensitive in related species, we studied four *C. briggsae* mutations orthologous to alleles of important *C. elegans* genes. Both *Cel-glp-4(bn2)* and *Cbr-glp-4(v473)* are temperature-sensitive, causing sterility at 25°C. By contrast, *Cel-fog-1* ( *q253)* is strongly *ts* , but its ortholog *Cbr-fog-1(v442)* causes a loss-of-function at all temperatures. Finally, the *C. elegans* *glp-1* alleles *bn18* and *e2141* are *ts* sterile. However, their *C. briggsae* orthologs, *Cbr-glp-1(v429)* and *Cbr-glp-1(v438)* respectively, are wild-type at all temperatures. Thus, a *ts* mutation in one species provides clues about how to design *ts* alleles in another, but all theoretical outcomes are possible.

**Figure 1. Analysis of temperature-sensitivity in orthologous mutations:**
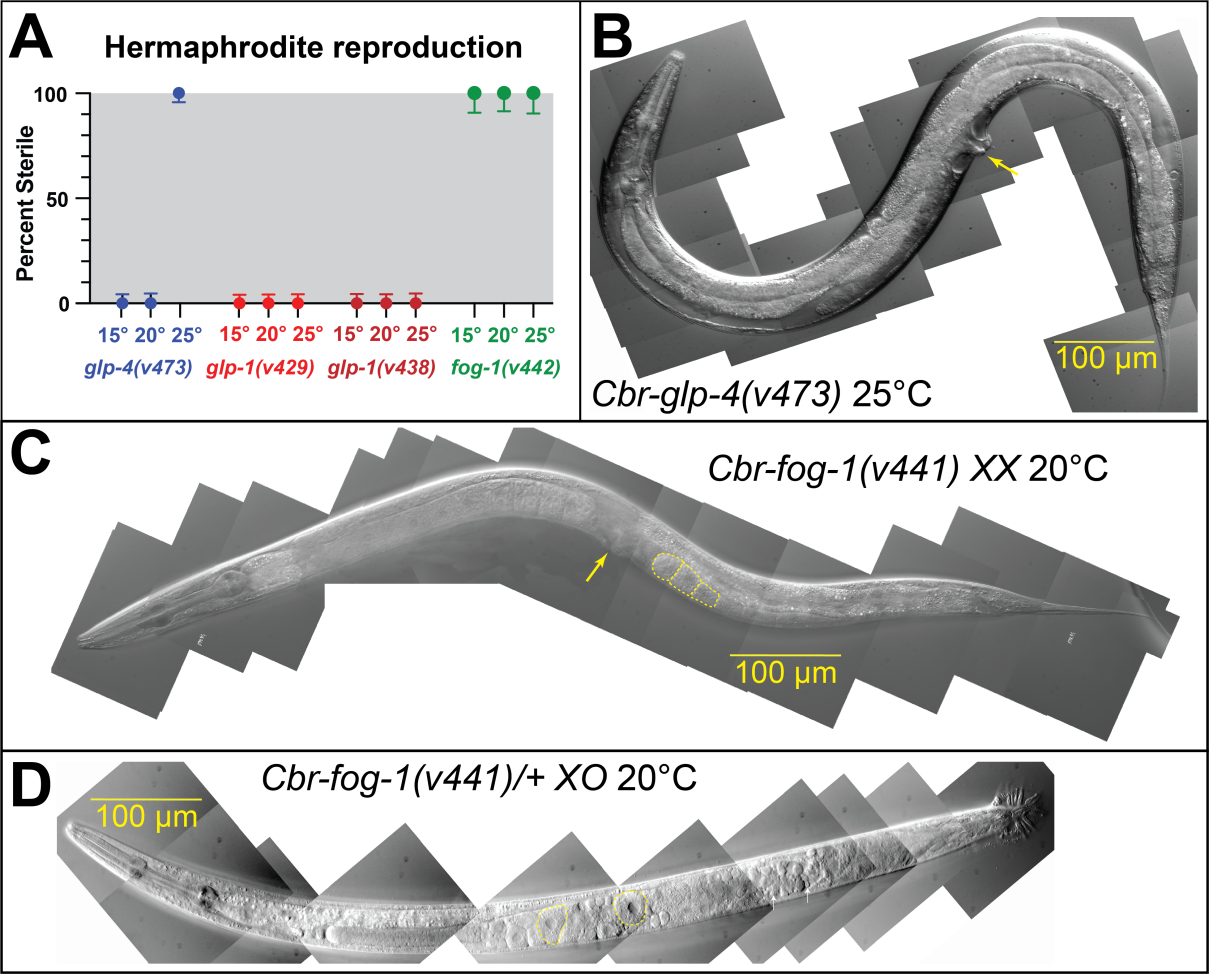
(A) Graph showing sterility of *C. briggsae* mutant hermaphrodites at three temperatures. Circles represent the percent displaying the trait, and lines represent 95% confidence limits, calculated using the Wilson/Brown method, implemented by GraphPad Prism. (B) Differential Interference Contrast (DIC) image of a *glp-4(v473)* *XX* animal at 25° C. The yellow arrow marks the vulva. There is no visible germ line. Anterior is left and ventral is down. (C) DIC image of a *fog-1(v441) XX* animal at 20°C. The yellow arrow marks the vulva. The uterus is empty and only oocytes are differentiating in the germ line. Three of them are outlined in yellow. (D) DIC image of a heterozygous *fog-1* *XO* male, showing oocytes outlined in yellow, as well as sperm (white arrows).

## Description

Temperature-sensitive mutations have a wild-type phenotype at the normal (or permissive) temperature and a mutant phenotype at the restrictive temperature, and are versatile tools for studying gene function. The temperature-sensitive proteins may exhibit altered stability, failure to fold or aggregate, resistance to proteolysis, or be cleared more quickly because of partial unfolding, since any of these factors might reduce function at unfavorable temperatures (Poultney et al., 2011). *C. elegans* has several *ts* alleles of critical genes that are helpful for genetic and developmental studies. To determine whether the temperature-sensitive behavior of mutations in *C. elegans* and *C. briggsae* is evolutionarily conserved, we constructed orthologs of four *C. elegans* germline *ts* mutations in *C. briggsae* using CRISPR-Cas9 (Farbound and Meyer, 2015) or TALEN (Wood et al., 2011, Wei et al., 2014) mediated gene editing.

The *glp-4* gene encodes a Valyl Aminoacyl tRNA Synthetase, essential for populating the germline with sufficient numbers of cells for gametogenesis (Rastogi et al. 2015). At the restrictive temperature of 25°C, *C. elegans glp-4(bn2ts)* mutants are sterile, because they produce very few germ cells, all of which arrest at meiotic prophase (Beanan and Strome 1992). By contrast, they are self-fertile hermaphrodites at the permissive temperature of 15°C. There is 87% sequence identity between Cel-GLP-4 and Cbr-GLP-4, and even higher amino acid sequence conservation (96%) within 50 amino acids of the *bn2* allele. This allele alters the Gly 296 residue to aspartic acid, and the orthologous CRISPR mutation in *C. briggsae* changes Gly 293 to aspartic acid. This ortholog, *C. briggsae* *glp-4(v473) I,* is also temperature-sensitive (Fig.1A); much like its *C. elegans* ortholog, it results in sterility and germ cells that fail to differentiate into gametes only when grown at 25°C (Fig. 1B). Thus, *Cbr-glp-4(v473ts)* provides a valuable new germline-proliferation-defective *ts* allele for *C. briggsae* .

FOG-1 is a Cytoplasmic Polyadenylation Element Binding (CPEB) protein that controls the sperm fate in *C. elegans* (Barton and Kimble, 1990, Luitjens et al., 2000, Jin et al., 2001a). In *fog-1* mutants, germ cells that would normally develop into sperm instead become oocytes. Furthermore, *fog-1* is required for spermatogenesis in both *XO* males and *XX* hermaphrodites. The *C. elegans* *fog-1* allele *q253ts* is a replacement of Thr 366 by Ile (Jin et al., 2001). It is strongly temperature sensitive, causing *XX* animals to become self-sterile at 25°C but not at the permissive temperature of 15°(Barton and Kimble, 1990). Although Cel-FOG-1 and Cbr-FOG-1 share only 54% sequence identity, they have higher conservation near the site of *q253* . The *Cbr-fog-1(v442) I* allele has an orthologous change (Thr 391 to Ile) that we made using TALEN gene editing. In contrast to *C. elegans* *q253ts* , the *Cbr-fog-1(v442)* allele causes a loss of function at all temperatures (Fig. 1A). The *XX* hermaphrodites made only oocytes (Fig. 1C) and the *XO* males produced oocytes at both permissive and non-permissive temperatures. As in *C. elegans,* this mutation is semidominant in males (Fig. 1D), which implies conservation of how *fog-1* is regulated in this sex. Since the homozygotes are self-sterile, we balanced *Cbr-fog-1(v442)* with *unc-40(v270)* .

GLP-1 is a notch receptor protein that regulates the mitotic proliferation of germ cells (reviewed by Kimble and Crittenden, 2007). The *C. elegans* *glp-1* alleles *bn18* and *e2141* are strongly temperature-sensitive, blocking germline proliferation at restrictive temperatures and causing sterility (Kodoyianni et al. 1992, Mello et al., 1994,). *C. elegans glp-1(e2141ts* ) is a missense allele that changes arginine 974 to cysteine, and is orthologous to *Cbr-glp-1(v438),* which we made using CRISPR/Cas9. Although the *C. briggsae* allele changes arginine 955 to cysteine, it is not *ts* , since the mutants appear wild type at both permissive and restrictive temperatures (Fig. 1A).

We also compared *Cel-glp-1* ( *bn18* ts) with *C. briggsae* . The *bn18ts* allele replaces alanine 1034 with threonine, and is orthologous to *Cbr-glp-1(v429),* an alanine 1020 to threonine substitution that we made using CRISPR-Cas9 *.* Unlike *bn18ts, Cbr-glp-1(v429)* is not sterile at either the restrictive or permissive temperatures, but instead develops like the wild type.

Although *C. elegans glp-1(ts)* alleles are sterile at the non-permissive temperature, neither of the *C. briggsae* orthologues showed sterility or defective germline development. However, a loss of function allele located nearby in the transcript does exhibit a Glp phenotype. *Cbr-glp-1(v439)* is a frameshift mutation caused by the deletion of nucleotide 2867 T in the coding sequence, near the site of *v438.* These mutant worms are sterile and display the Glp-1 defective phenotype at all temperatures (Rudel and Kimble, 2001). Thus, the *glp-1* loss-of-function phenotype is conserved between *C. briggsae* and *C. elegans* but not the temperature-sensitivity of key alleles.

Taken together, our findings show that one of the four *C. elegans* *ts* alleles we studied had similar behavior in *C. briggsae* . However, the other alleles were only *ts* in one species. Thus, mutations orthologous to known *ts* alleles can be temperature-sensitive in other species, and provide a promising guide for generating such alleles, but their behavior often differs. Although high levels of structural conservation might suggest that mutations will behave similarly in both species, we found it hard to predict which alleles will be *ts* without experimental tests. We suspect that *C. elegans* and *C. briggsae* sometimes differ because some genetic backgrounds are more sensitive to perturbation than others.

## Reagents

**Reagents**

**Table:** 

**Strain**	**Genotype**	**Phenotype**	**Availability**
CB4037	*glp-1(e2141) III*		CGC
DG2389	*glp-1(bn18) II* .		CGC
SS104	*glp-4(bn2) I*		CGC
JK560	*fog-1(q253) I*		CGC
RE1206	*Cbr-glp-1(v438) III*	wildtype	Ellis Lab
RE1208	*Cbr-glp-1(v439)/ Cbr-lin-39(bh20) III*	Glp	Ellis Lab
RE1186	*Cbr-glp-1(v429) III*	wildtype	Ellis Lab
RE1274	*Cbr-glp-4(v473ts) I*	TS Glp	Ellis Lab
RE1229	*Cbr-fog-1(v442)/Cbr-unc-40(v270) I*	Fog	Ellis Lab
